# Hand-Fed Children

**Published:** 1888-12-08

**Authors:** 


### Within the Wards.

### HAND-FED CHILDREN.

At the recent annual meeting of the British Medical Association there was an important discussion on "Rickets." A distinguished physician read a paper at the outset. One passage of that paper parents and medical men everywhere will do well to lay to heart. The speaker said: "One of the first and best established facts with regard to the food connexion is that rickets is almost entirely confined to *hand-fed children.* Children at the breast escape, whatever the other hygienic conditions may be, at any rate until after weaning. In rare instances, if the mother is greatly enfeebled, rickets may develop, yet the only example which I can call to mind of rickets arising in a child suckled by a healthy mother is one in which the mother became pregnant while suckling. . . . The next baby was born healthy and remained so. The child at the breast developed marked rickets." Many people consider rickets a mere affection of the bones. Even if it were, it is an eminently unsatisfactory disease. The deformities of rickety people are not only unsightly to others, but must be both distressing and disabling to those who have them to endure. The reader of the paper insists that the disease "is not to be regarded as a mere affection of the bones. This (the bone affection) is so obvious and striking a feature of the nachitic state that it has assumed undue importance, and rickets has been in danger of being looked upon as simply a defective and perverted development of the osseus structures, and has, indeed, been thus defined quite recently by a leading medical authority. But it is something more than this. The disease affects not only bones, but muscles and ligaments, mucous membrane, and skin, the blood and the nervous system." This view of the case is undoubtedly correct. Rickets is a disease of the whole physiological economy. It is, morever, a disease which is in a high degree preventable; and its prevention is to be effected by measures of the simplest and most natural kind. If mothers could suckle their own children, rickets in early infancy would be comparatively unknown. But the very fact that natural feeding in infancy is an almost certain preventive of rickets justifies the further inference that natural feeding in childhood and youth will lead to a similar result. Nay, it points to an even larger inference, viz., that natural feeding throughout life would probably prevent many other constitutional diseases as well as rickets. Natural food for infants, according to this observer, must include milk, raw meat, or cream. A purely farinaceous diet is a fruitful source of rickets. If this be so in the case of mere babies, how much more necessary must it be that older children and grown-up people should have a diet which includes meat in due proportion? Vegetarianism, pure and simple, is probably not the dietary of the future.

